# Supplementary material for: Are carbon emissions trading and green financial instruments synergistic? -Comprehensive quantitative research based on content analysis
Source: PLoS One. 2024 Mar 7;19(3):e0298601. doi: 10.1371/journal.pone.0298601 (PMC10919601; doi:10.1371/journal.pone.0298601)
Supplement: S1 Data — (ZIP) [file pone.0298601.s001.zip › Data/地级市Z_F工作报告.zip!/║┌┴·╜¡/║┌║╙╩╨/└┤╫╘╣1⁄2╓┌║┼í╛┬φ┐╦╩2╛▌═°í┐_2017.docx]

各位代表：

现在，我代表市人民政府向大会作工作报告，请予审议，并请列席会议的同志提出宝贵意见。

一、过去五年和2016年工作回顾

过去五年，政府工作在省委、省政府和市委正确领导下，在市人大、市政协监督支持下，紧紧依靠全市人民，主动适应经济发展新常态，找准定位、积极作为、精准发力，经济社会实现跨越式发展。预计地区生产总值增长49.6%，年均增长8.4%。全社会固定资产投资增长93%，年均增长14.1%。社会消费品零售总额增长79.5%，年均增长12.4%。公共财政收入增长59.4%，年均增长9.8%。经济总量由全省第9位晋升到第8位。这五年，是我市综合实力显著提升、人民生活持续改善、社会保持和谐稳定的五年，是奋进的五年、辉煌的五年。坚持开放发展理念，集中力量打基础、补短板，寄托黑河人民28年夙愿与梦想、在中俄区域合作中具有里程碑意义的黑龙江大桥正式开工，极大地提升了黑河在“一带一路”与欧亚经济联盟对接中的战略地位。中俄东线天然气管道开工建设，为深化黑河与阿州能源合作开辟了新的广阔空间。五大连池德都机场当年施工、当年完成主体工程，为旅游产业发展创造了有利条件。“三江四岛”工程功在当代，利在长远，开创了黑河水利建设史上的新纪元。坚持绿色发展理念，把良好的生态作为最宝贵财富，加大保护开发力度，全市整体纳入大小兴安岭重要森林生态功能区。环境质量位于全省前列，被评为“中国十佳空气质量城市”，绿水青山、蓝天白云成为黑河一张靓丽的名片。坚持创新发展理念，推动供给侧结构性改革，发展寒地生态农业，被评为国家“粮食生产先进市”。把矿产资源开发作为强市富民的支柱产业，建成了多宝山铜矿等一批牵动力强的重大项目。推动互联网与传统产业融合，跨境电商成为经济发展新引擎。中俄文化大集上升为国家级活动，促进了经济互补、情感互融、民心相通，开创了中俄文化交流的新模式。旅游文化、跨国旅居养老产业异军突起，黑河正成为国内外游客休闲养生的新热点。坚持协调共享发展理念，加大民生投入力度，群众生活水平明显提高，居住条件大幅度改善，社会事业全面进步。凝聚顽强拼博精神力量，战胜2013年特大洪涝灾害，保护了人民生命财产安全。荣获国家卫生城、国家园林城称号，国家环保模范城正待验收，获得全国文明城提名，荣获“全国首批民生改善十佳典范城市”。军政军民融合发展，荣获全国双拥模范城“三连冠”。

回首过去的五年，成绩来之不易，奋斗充满艰辛。2016年，我们着力稳增长、调结构、促改革、惠民生、防风险，实现了“十三五”良好开局。预计地区生产总值490亿元，增长6.5%。全社会固定资产投资275亿元，增长8%。社会消费品零售总额114亿元，增长10%。公共财政收入突破30亿元大关，增长4.7%。城镇常住居民人均可支配收入24765元，农村常住居民人均可支配收入12968元，分别增长8%和6.5%。万元地区生产总值综合能耗下降5%以上。

(一)狠抓产业项目，集聚经济发展动能。省市级重点产业项目开复工率和投资完成率均达到90%以上。加快完善园区功能，一批大项目入驻，黑河(北安)健康产业园创造了全省园区建设新速度。开展“招商之冬”活动，广泛宣传推介，招商引资落实项目196项，实际到位资金217亿元，增长7.6%，其中新建超亿元项目29项，实际到位资金26.6亿元。创新投融资方式，与深圳前海两型金融控股集团等基金公司合作，设立了百亿元城市发展基金和多支子基金，首期募集规模20亿元，金融支持地方经济发展的能力进一步增强。成立企业上市孵化基地，金禾农业、红河谷试车公司在新三板挂牌，骏通经贸在新四板挂牌，恒灿旅游在上海股权托管交易中心E板挂牌。强化与社会资本合作，谋划交通设施、市政设施、公共服务等重点领域项目35个，全部纳入省PPP项目库。

(二)推动现代农业发展，提升综合生产能力。紧盯市场需求，科学调整种植结构。推动大豆产业振兴，“黑河大豆”地理标识在国家商标局备案注册，我市被命名为中国绿色生态大豆生产示范基地、中国优质大豆生产基地。大力发展青贮玉米、水稻和汉麻、饲草等优质高效作物，孙吴被命名为“中国汉麻之乡”。加大科技推广力度，建设农业科技示范园区77个。实施畜牧业发展三年规划，新建标准化牧场16个。发展林业特色产业，北药累计种植面积63万亩，小浆果繁育面积11.2万亩，林业产值增长24%。发挥龙头企业示范带动作用，全市规模以上企业农产品加工交易130万吨以上，实现销售收入36亿元。提高现代农机合作社建设经营水平，新建现代农机合作社16个，创建省级规范社7个。积极培育新型农业经营主体，带动土地规模经营1200万亩。孙吴桦林现代农机专业合作社吴德显被评选为第二届“全国十佳农民”，是东北地区唯一获此殊荣的农民。

(三)融入沿边开放大局，全面深化对俄合作。全市对外贸易进出口总值实现5.7亿美元。进一步调整贸易结构，扩大粮食、木材、电力等商品进口规模，对俄进口企稳回升。加强投资合作，油品储运与炼化综合体、水泥粉磨站项目列入俄超前发展区重点支持名录。境外汽车服务平台、木材加工厂建成使用。境外开发土地达到126万亩。合作区利源达公司专用车辆出口加工项目一期试生产。境外石化建材加工和农业产业园区入驻企业10家，阿州工业园区孵化中心建成。保税物流中心(B型)开工建设。优化口岸环境，新建旅检入境大厅投入使用。黑河口岸成为我省首批境外旅客离境退税试点。做强互市贸易，大黑河岛边民互市贸易监管点储存仓库完成改造，逊克互贸区海关监管库、民贸交易大厅完工。搭建经贸平台，成功举办中俄边境城市展览会，扩大了知名度和影响力。

(四)发展新兴产业，培育经济新增长点。加快旅游产业发展，黑河、五大连池被国家确定为“全域旅游示范区”创建单位。“静静的黑河”等3条线路入围全省首届旅游产品设计大赛并获奖。“龙江之星”旅游专列开通，旅游宣传片覆盖全国高铁车组。五大连池风景区被国家旅游局命名为中国首批康养旅游示范基地，荣获中国首个世界生物圈保护区管理最高奖——米歇尔·巴蒂斯奖，作为东北地区唯一主会场，成功举办了全国新年登高健身大会。预计全市接待国内外旅游者805万人次，旅游收入64.5亿元，分别增长23.9%和22.2%。推动文化产业发展，中国书画艺术院黑河分院开工，《两个人的山林》《火线出击》等影视剧在黑河拍摄。突出冰雪资源特色，举办了2016“冰雪童话快乐之冬”主题乐园暨首届中俄冰雕雪雕大奖赛。培育养老产业，五大连池风景区—黑河—布市“两国三地”中俄跨国旅居养老成为我省7条精品线路之一，候鸟旅居养老突破3万人次。试车经济如火如荼，宝马、奔驰、奥迪等99户企业入驻黑河进行寒地试车，国内整车生产企业寒区试验量占全国80%以上。珠宝玉石年交易额突破2亿元。

(五)完善城市功能，提升城市形象。启动海绵城市和城市综合管廊建设规划编制。电业街、江天路、桦林路、魁星路、民益街等道路延伸和修复工程全部完工。启动大黑河岛绿化恢复、黑龙江沿江公园改造和体育公园、中俄双子城公园建设，完成市城区22条街路绿化、14条街路绿化补植和53个小区架空管线归集整理。市城区改造建设供水管网5.2公里，新建供热管网22.8公里，改造老旧供热管网15.7公里。城市管道燃气工程开工建设，铺设燃气管网10公里。提升智能化管理水平，建立了24小时城市动态监控、应急响应管理运行机制。完善各县(市)城市功能，北安祥和公园市民广场基本完工，嫩江城东垃圾处理场全面完工，五大连池滨水新城道路等基础设施全部开工，孙吴秀河公园启动建设，逊克滨江路改造加快实施。坚持新型城镇化与农业现代化同步发展，完善15个试点镇基础设施，打造美丽宜居乡村，村容村貌明显改观。

(六)坚持共建共享，着力改善民生。加大脱贫攻坚力度，37个贫困村实现整村推进，75个扶贫项目全面实施。大力推进保障性住房建设，全市棚户区改造开工1701户。农村泥草(危)房改造开工5208户。积极解决市城区房屋办证遗留问题，涉及居民6700余户，面积65万平方米。鼓励创新创业，全市城镇新增就业16029人，30周岁以下高校毕业生就业1836人，城镇登记失业率3.59%。首次开展了中俄知识产权文化交流活动。建立科技企业孵化器和众创空间7家，黑河市创客空间通过国家科技部首批备案。深化人才发展体制机制改革，出台激励人才创新创业实施办法，引进急需紧缺高层次人才28人，培养高技能人才512人。统筹各类教育协调发展，教育信息化水平居全省前列。新建改扩建公办幼儿园8所，创建义务教育标准化学校7所，五大连池省部共建国家现代农村职业教育改革试验区试点县、国家级农村职业教育和成人教育示范县通过验收。深入推进第四批公立医院综合改革，市第一人民医院与复旦大学附属中山医院合作成立医疗技术协作中心，建立中俄友好医院。群众体育蓬勃开展，竞技体育稳步提升，全民健身中心体育馆主体完工。爱辉区被国务院授予“全国民族团结进步模范集体”称号。机关事业单位养老保险制度改革全面推开，城镇职工(居民)基本医疗保险实现市级统筹，城乡居民大病医疗保险制度启动实施。强化食品安全监管，打造餐饮服务“明厨亮灶”153家。建立综合监管信息平台，安全生产形势保持稳定。连续21个防火期未发生重特大森林火灾。积极化解信访积案，解决群众合理诉求，信访形势总体平稳。推进“平安黑河”建设，社会治安防控体系进一步完善，荣获“全国法治宣传教育先进城市”称号。老区、防震减灾、侨务、妇女儿童、人防、档案、地方志等工作取得新成绩。

(七)提高行政效能，加强政府自身建设。深入开展政府系统“两学一做”学习教育，突出问题导向，全力进行整改。开展专业知识专题学习活动，提升政府工作专业化水平。主动接受人大、政协监督，办理建议和提案155件。完善市政府重大决策制度，实施社会稳定风险评估32项，重大决策合法性审查率100%。推行“双随机一公开”，行政执法事项全部纳入抽查范围。推进行政审批制度改革，统一规范行政权力3946项，取消和规范证明事项83项。深化商事制度改革，全面启动企业“五证合一”和个体工商户“两证整合”工作。开通了“信用黑河”网站，建立了社会信用体系基本框架。市行政服务中心综合大厅投入使用。市公共资源交易中心办理交易工程项目106项，节支率14.6%，完成政府集中采购项目38项，节支率8.6%。发挥企业投诉中心作用，办理企业投诉15件、优化发展环境案例19件，问责28人。强化行政监察和审计监督，廉政建设进一步加强。

各位代表！过去的五年，全市经济社会发展取得了显著成绩，这是省委、省政府和市委正确领导的结果，是市人大、市政协和社会各界鼎力支持的结果，是全市人民团结一致、共同奋斗的结果。在此，我代表市人民政府，向全市各级人大代表、政协委员和各民主党派、工商联、无党派人士、人民团体，向中省直各单位和人民解放军、武警官兵，向全市人民，表示衷心感谢，并致以崇高敬意！

我们也清醒地认识到所面临的困难和问题：经济下行压力较大，主要经济指标增长乏力；主导产业尚未形成，产业结构不够合理，结构调整任务艰巨；主动对接“一带一路”意识不强，区位优势发挥不够，对俄合作转型升级缓慢，区域中心城市集聚和辐射作用尚未显现；对人才的吸引力不够，人才结构不尽合理，特别是高层次人才外流还没有从根本上改变；思想解放程度不够，对经济新常态认识不足，把握引领新常态的能力亟待提升；个别干部担当精神不够，不作为、慢作为、不敢为的现象依然存在。这些问题和不足，我们必须高度重视，切实加以解决。

二、今后五年奋斗目标

今后五年，是全面建成小康社会、实现第一个百年梦想的冲刺期，也是实现黑河全面振兴的关键期。纵观发展大势，国内产能过剩和需求结构性矛盾仍然突出，我市也面临着快速扩大总量和提高发展质量的双重困难。在面临严峻复杂外部环境的同时，我们正恰逢难得的发展机遇。党中央、国务院高度重视东北和沿边地区发展，确立了老工业基地振兴和沿边重点地区开发开放政策框架，我市纳入国家顶层设计，政策效应叠加释放，战略地位更为凸显。习近平总书记系列重要讲话特别是针对龙江两次重要讲话，使我们在全国全省大格局中，认清了所处的历史方位和肩负的职责使命，明晰了振兴发展的方向和路径。中俄全面战略协作伙伴关系步入新阶段，黑河与阿州作为两国最大毗邻地区，跨境发展要素高度聚合，必将成为“一带一路”与欧亚经济联盟对接的区域中心和重要支点。经过历届班子不懈努力，我市综合实力持续增强，产业基础更加坚实，得天独厚的区位条件和资源禀赋，使我们在对俄、生态、旅游、养老等国内总需求有增长空间的领域，有突出的比较优势和供给优势，为今后发展积蓄了强劲动能。面对新形势、新任务、新挑战，我们必须准确把握发展大势，借助国家振兴东北老工业基地、深化沿边开放等一系列重大战略的东风，谋势而动、蓄势而为、乘势而上，在全面建成小康社会的伟大征程中承担起神圣使命，在推动黑河全面振兴的生动实践中建功立业。

今后五年，我们要按照市第六次党代会要求，高举中国特色社会主义伟大旗帜，以马克思列宁主义、毛泽东思想、邓小平理论、“三个代表”重要思想、科学发展观为指导，深入学习贯彻习近平总书记系列重要讲话精神和对黑龙江工作的重要指示，认真落实省委部署，统筹推进“五位一体”总体布局，协调推进“四个全面”战略布局，牢固树立和贯彻落实五大发展理念，以解放思想为先导，以提高质量和效益为中心，以供给侧结构性改革为主线，坚持产业拉动、开放带动、创新驱动，充分发挥比较优势，大力构建“十大新体系”，打造黑河发展的新格局，确保全面建成小康社会目标如期实现，为黑河全面振兴打下更加坚实的基础。

刚刚闭幕的市第六次党代会，确定了今后五年的奋斗目标：到2018年，提前实现全面建成小康社会关键指标，地区生产总值和城乡居民人均收入比2010年翻一番。到2020年，圆满完成“十三五”规划任务，打赢脱贫攻坚战，如期实现全面建成小康社会目标。到2021年，力争地区生产总值突破1000亿元大关，城乡居民人均收入分别突破35000元和20000元，黑河发展登上一个新台阶。

——今后五年，我们将倾力打造对外开放大格局。始终站在国家和全省对外开放的战略高度，全力争取国家支持，设立跨境经济合作区、沿边开发开放试验区、跨境旅游合作区和边境旅游试验区、沿边金融综合改革试验区，加快建设黑龙江大桥桥头区、黑河保税物流园区，构筑中俄边境地区全新开放合作平台，使黑河与阿州成为中俄边境区域要素跨境流动、产业深度对接的经济共同体，在更大范围、更广领域、更高层次上融入东北亚区域合作，引领沿边开放转型升级。

——今后五年，我们将倾力畅通国际物流大通道。积极融入“一带一路”“龙江丝路带”整体布局，建成黑龙江大桥和跨江空中索道，完善黑河、逊克、孙吴口岸功能，开通黑河国际航空港，建设国际陆海空大通道，全面建成面向俄罗斯、连接亚欧的综合跨境运输通道，对内辐射全国，对外辐射俄远东和腹地城市、日韩、东南亚等地区，打通黑河走向世界的大动脉。

——今后五年，我们将倾力推进经济发展跃上新台阶。牢牢坚持新发展理念，积极探索新发展路径，扬长克短、扬长避短、扬长补短，充分发挥重大项目引领作用，力争转变发展方式取得重要进展，传统优势产业强筋健骨，战略性新兴产业蓬勃兴起，现代服务业成为新增长点，新型工业化、信息化、城镇化和农业现代化水平不断提高，全力构建“十大新体系”，保持经济增速高于全省平均水平，综合实力排名在全省实现晋位赶超。

——今后五年，我们将倾力谱写城市文明新华章。坚持绿色循环低碳理念，顺应城市发展规律，体现历史传承、地域文化、时代要求，完善城市功能，打造城市风格，延续历史文脉，建设欧陆风情园林城市。凝练城市精神，创建全国文明城市，使城市风格与服务功能内外兼修，城市气质与市民素质齐头并进，提高城市发展的持续性、宜居性，让城市生活更美好。

——今后五年，我们将倾力让群众生活更殷实。民生无小事，民心是大事。我们要把更多的财力物力向民生倾斜，关注群众的衣食住行，心系百姓的安危冷暖，城乡居民收入大幅增长，劳动就业更加充分，社会事业持续繁荣，社会保障全民覆盖，让底线更牢固，生活更便利，出行更顺畅，日子更红火，红利全民共享。各位代表，宏伟目标已经明确，美好愿景催人奋进。我们坚信，在市委坚强领导下，通过各级广大干部和全市各族人民共同努力，全面建成小康社会的目标一定能够如期实现！

三、2017年目标任务

2017年是新一届政府开局之年，我们肩负着推进黑河全面振兴的新使命，承载着全市人民对美好生活的新期盼。我们要始终坚持稳中求进这个总基调。深刻理解党中央关于经济发展进入新常态的重大判断，把握“稳”这个基础，做足“进”这篇文章。“稳中求进”对黑河来讲，重点在“进”，核心在“进”。要在“进”上发力，扎实做好“三篇大文章”，挺过产业转型阵痛期，加快新旧动能转换，实现更高质量、更大效益发展。牢牢把握创新发展这个总要求。深入落实创新驱动发展战略，进一步创新思想观念、发展模式和工作机制，以创新增强动力、引领发展。一方面，要进一步解放思想，全力破除小富即安、思维固化、机制不活的思想束缚。另一方面，要深入推进供给侧结构性改革，提高供给质量，在国内外有总需求增长空间、黑河有鲜明供给优势的领域，创新升级产业链、价值链，抓住“两座金山银山”带来的绿色食品、生态旅游、冰雪经济、林产开发的潜力释放，加快培育新经济、新模式、新业态，使我们的优势资源尽快转化为产品和服务。紧紧扭住实体经济这个总抓手。实体经济是支撑经济发展、赢得竞争优势的根基。一方面，要优化存量，推进“三去一降一补”，防范重点领域风险，用新技术改造提升传统产业，推动资源精深加工，促进传统产业转型升级。另一方面，要做大增量，按照“五个要发展”“五头五尾”路径推进重大产业项目建设，发挥好产业项目对经济增长的支撑作用，精心培育壮大主导产业，打牢实体经济根基。时刻围绕改善民生这个总导向。保障改善民生没有终点，只有连续不断的新起点。一方面，要补齐民生短板，坚持守住底线、突出重点、完善制度、引导舆论，突出解决好人民群众的“难心事”“烦心事”。另一方面，要顺应群众新期盼，在全市财力有限的情况下，加大投入力度，努力为群众多办看得见、摸得着、有温度的好事实事，说一件干一件，干一件成一件，一步一个脚印，持续发力，久久为功。市政府将发布“民生工作报告”，梳理出民生工作具体任务，压实责任、提实要求、抓实考核。各级政府要始终直通人民群众，时刻将“以人民为中心”作为履职尽责的第一追求，让“全心全意为人民服务”成为各级政府的鲜明坐标！

今年，我们要认真贯彻中央和省委经济工作会议精神，落实省委、省政府领导指示，按照市第六次党代会部署，坚持统筹兼顾，突出重点，努力推动经济社会健康发展，为全面振兴打下坚实基础。全市经济社会发展主要预期目标是：地区生产总值增长6.5%以上，全社会固定资产投资增长8%，社会消费品零售总额增长10%，公共财政收入增长5%，对外贸易进出口总值增长3%，城镇、农村常住居民人均可支配收入均增长8%以上。着重做好八个方面工作：

(一)激发内生动力，培育壮大振兴发展新动能。将产业项目建设作为“一号工程”，培育特色产业，提供有效供给，为经济发展注入强劲动力。全市安排重点产业项目92项，其中，续建和新开工项目64项，年度投资85.3亿元，推进前期28项。总投资10亿元以上项目13项，总投资5～10亿元项目8项，总投资1～5亿元项目27项。做好“三篇大文章”。坚持创新驱动，在优化存量基础上做大增量，加快优势潜力释放，构建工业新体系。深度开发“原字号”，坚持“粮头食尾”“农头工尾”，培育壮大龙头企业，拉长大豆、玉米等产业链条，重点推进黑河大豆食品园、五大连池非转基因大豆加工、爱辉区非转基因大豆种植加工、孙吴天然汉麻纤维加工等项目建设，实现农产品多次加工、梯次增值、吃干榨净，增强市场竞争力。培育壮大“新字号”，抢占科技制高点，加快新中新物联网等新一代信息技术产业项目建设，推进新能源汽车、光伏发电、中药饮片、菲汀加工、废旧矿物油综合利用等项目落地。改造提升“老字号”，深度开发矿产资源，重点推进嫩江多宝山铜矿二期、逊克东安岩金矿、爱辉区振兴煤矿扩能改造等项目建设，提高矿业对经济发展贡献率。推进项目投产达效。严格落实包保责任，强化协调服务，全力推进省市重点产业项目建设，确保项目开复工率和投资完成率均达到90%以上，争取北安桦树茸加工、五大连池科迪稻谷加工等项目建成投产。鼓励现有企业技术改造，帮助北大荒矿泉水、盛辉林板一体化、嵩天薯业、金湾水电站、三精制药等企业和项目走出困境。推进500千伏黑河换流站增容及新一轮农网改造升级工程，为项目建设提供电力保障。加快园区整合，推进差异化发展，加大黑河进出口加工产业园、黑河(北安、孙吴)健康产业园、五大连池生态矿泉示范基地、逊克经济开发区等园区建设力度，增强产业集聚功能。探索建立项目退出机制，以土地清理倒逼项目投资，盘活土地资源，通过“腾笼换鸟”引入新项目，提高园区产出效益。积极推动金融创新。打好政策组合拳，充分发挥产业基金引导作用，深化与深圳前海两型金融控股集团等企业合作，撬动社会资本支持项目建设，破解融资难题。探索采取设立土地银行等现代融资手段，加快恒灿旅游公司融资进程。抓好上市企业后备资源库建设，推动温馨食品、长乐山大果沙棘等企业挂牌上市。争取将棚户区改造等一批项目纳入国家专项建设基金支持范围。加强与社会资本合作，围绕基础设施和公共服务领域，谋划储备实施一批PPP项目。开展精准招商。树立“大招商、招大商”理念，深入开展“招商之冬”活动，抓住发达地区加快产业梯次转移的机遇，以长三角、珠三角、京津冀为重点，紧盯国内外500强企业，开展“一对一”“点对点”宣传推介，力争招商到位资金242亿元，增长20%，落地超亿元项目30项。利用中俄博览会、龙港经贸合作交流等大型展会平台，捕捉投资线索，全程跟踪服务，推动项目尽快落地。做强县域经济。坚持站在全市高度统筹谋划，各县(市、区)要根据资源禀赋、产业基础，找准比较优势，明确主攻方向，纳入市级考核体系，努力形成特色鲜明、优势互补的县域经济发展格局。北安、嫩江要当好县域经济的排头兵，助推全市经济快速发展。

(二)破解发展瓶颈，推动重大基础设施建设。发挥基础设施建设在引领经济转型升级中的关键作用，着力补齐发展短板，形成支撑黑河发展的有力保障。构建交通新体系。推进跨境设施联通，加快黑龙江大桥建设，跨江空中索道开工，同步启动桥头区和口岸联检设施建设，修订市城区功能区规划，研究谋划“大桥经济”。争取黑河国际空港早日开通对俄空中航线，确保五大连池德都机场和嫩江通用机场年内通航，加快形成“空港经济圈”，带动区域经济发展。推进北黑铁路资产重组和升级改造工作，争取北五铁路、黑河至乌伊岭沿边铁路开工建设。推进嫩江至双辽公路项目，加快国铁富嫩线电气化升级改造进程。加强水利设施建设。实施水电、农田水利、河流治理、水库除险、农村饮水安全巩固提升工程等项目16项，提升水利基础保障能力。完成岸岛防护三期规划审批，建设101公里防护工程。编制黑龙江黑河水利风景区总体规划，做好省级水利风景区申报工作。加大保障性安居工程建设力度。打好市城区棚户区改造攻坚战，开工建设西部和西南部6个棚改项目，同步完善道路、绿化、供水、供热、供气、供电等配套设施，力争年内市城区棚户区基本消除。购买公共租赁住房150套，发放租赁补贴5000户，改造农村泥草(危)房3700户，让群众住得更安全、更温暖、更舒心。

(三)推进供给侧结构性改革，建设现代农业强市。主动适应农业发展新变化，使好资源开发出好产品，好东西卖出好价钱，构建现代农业新体系。发展绿色优质生产。把增加绿色优质农产品供给放在更加突出位置，根据市场对优质、营养、安全农产品的需求，调整种植结构，增小麦、减玉米、调控大豆、大力发展高效经济作物。做大做强大豆产业，推进食品豆专用品种生产基地和非转基因大豆生产保护区建设。推广强筋春小麦优质新品种，建设优质特色生产基地。扩大青贮玉米、专用玉米面积。大力发展汉麻产业，开展玫瑰、赤芍等花卉种植及产业链研究，扩大沙棘、蓝莓、北药等特色种植规模。引导农业发展向绿色并轨，推广蟹稻、鱼稻等绿色生态技术模式。全市绿色有机农产品认证数达到85个，面积扩大到330万亩，增长13%，认证地理标识农产品12个。强化农产品质量监管，组织农业生产合作社、加工企业进驻省农产品质量追溯体系，抓好嫩江农产品质量安全示范县建设，为群众吃上放心粮、放心菜提供保障。大力发展畜牧业。将畜牧产业培育成为振兴发展的战略性产业，构建畜牧产业新体系。积极发展“两牛一猪一马”和寒地特色养殖，实现粮变肉、草变乳过腹增值。推行“标准化集中饲养+适度规模养殖场”模式，培育养殖大户、家庭牧场、养殖合作社和龙头企业，加快建设高品质奶牛、安格斯肉牛、生猪和黑河挽马产业基地，推进温氏畜牧一体化生猪养殖等项目。培育壮大中兴牧业、宜品乳业、鑫宇牛业等龙头企业，按照“公司+家庭农场”合作模式，引导龙头企业与农民构建产业共同体，带动农民增收致富。加快产业化进程。主动适应产业分化延伸新特点和消费升级新需求，加快一、二、三产融合。支持新型经营主体与企业合作，联合组建大豆、小麦、山特产品等产业集团，提高农林产品加工转化率和增值率。扩大爱辉区坤河现代农业基地、五大连池农业观光采摘园、嫩江农业科技园区规模，培育农业采摘、乡村游等新业态。大力发展生态经济林、北药、森林绿色食品、生态旅游、种苗繁育、林下特色养殖六大林业产业，力争林业产值增长10%、林下经济产值增长15%。提升适度规模经营水平。把现代农业合作社作为适度规模经营主攻方向，抓好黑河大豆种植和北安城东现代农业等7家联合社建设，新建现代农机合作社10个，创建省级规范社5个，市级规范社10个，实现生产全程机械化。推进土地制度改革，完成集体耕地确权登记。推进孙吴农村土地银行试点，探索降低耕地使用成本和风险新机制。做强圣丰种业、富民种业，提高优质良种统供率。创新农业保险产品，扩大覆盖面，提高农业抗风险能力。加快农业科技创新。引进高端人才，建设大豆、小麦、水稻、黑河挽马院士(专家)工作站，开展大豆、小麦优质高产高效攻关。完善提高3个“互联网+”农业综合服务大厅、3个“互联网+”农业示范基地和71个现代农业科技园区建设水平，推广优质高效增产技术，农业科技贡献率达到53%以上。强化市场营销。集中使用“黑河大豆”地理标识，加大“北纬49度”“硬红春小麦”“非转基因大豆”“嫩江赤芍”等品牌宣传力度，提升地方产品价值链。将品牌建设与网上营销相结合，做大做强北安村淘、嫩江聚超网、爱辉区惠丰通村网、五大连池苏宁电商试点项目，培育一批电子商务特色村和农村电商示范企业，完善嫩江非转基因大豆物流交易中心、孙吴山珍产品交易市场，做大黑河绿色食品专柜旗舰店，推动农产品由“种得好”向“卖得好”转变。

(四)拓展对俄合作，提升外向经济水平。主动融入“一带一路”“龙江丝路带”建设，加快打造平台坚实、产业多样、内外衔接的对俄合作新体系，全力推动对外开放战略升级。扩大贸易领域。巩固主营进出口商品规模，稳定果蔬、农产品、工程机械出口，增加风电设备、硅基材料、木制品等地工产品出口。扩大电力、木材、粮食进口规模，增加俄优质特色商品进口种类。支持新型贸易发展，以跨境电子商务为引领，推动对俄贸易转型升级，完善合作区跨境电商产业园区功能，争取创建国家级电子商务示范基地，支持中机电子公司为代表的跨境电商平台建设，完善支付、通关、物流、结汇、退税等配套服务体系，力争交易额达到7亿元，增长20%以上。繁荣互市贸易，完善具体政策措施，争取放宽互市贸易商品进口品种。加快项目建设。坚持“出口抓加工、进口抓落地”，吸引和服务国内企业参与俄资源能源开发。开工油品储运与炼化综合体项目，推进萨哈(雅库特)共和国油田区块勘探开发。以对俄农业种植企业为主体，在俄境内建设大豆加工厂，生产膨化大豆粉、卵磷脂等产品，年加工能力达到10万吨以上。扩大境外森林采伐加工规模，年采伐加工30万立方米。搭建合作平台。完善黑河进出口加工产业园功能，壮大专用车、硅硼新材料等产业规模，培育保税物流加工、能源装备制造等新兴产业。加快境外石化建材、中俄农业产业、阿州工业园区建设，争取纳入省对俄境外园区发展规划。抓住阿州超前发展区政策机遇，引导更多中方企业和项目入驻，鼓励企业投资向俄腹地城市延伸。黑河保税物流中心(B型)主体工程完工。开工建设黑河口岸物流园区。加强人文交流。密切黑河与阿州布市两地高层交往、互访和部门间合作，加强与俄腹地城市交流，丰富对俄民间交往载体。探索以市场化机制办好第八届中俄文化大集，与俄方共同举办好中俄边境城市展览会、阿州国际洽谈会、中俄企业项目推介会和中俄林业生态建设合作论坛。成立国际商会法律专业委员会，为企业“走出去”提供更好服务。

(五)厚植产业新优势，培育壮大新兴业态。发挥好“绿水青山、冰天雪地”的资源优势，把开发利用“两座金山银山”作为创新升级现代服务业的重要依托，构建服务业发展新体系。打造旅游支柱产业。把旅游产业作为促转型调结构稳增长的首位产业，以市城区和五大连池风景区为两大核心，加快构建全域旅游新体系，推进黑河全域旅游示范区建设。全年接待国内外旅游者突破900万人次大关，旅游收入达到76亿元。加快瑷珲乡野公园、五道豁洛养生岛等旅游项目建设，提升景区景点品质。争取国家支持，推进跨境旅游合作区和边境旅游试验区在黑河先行先试，争取自驾车出入境旅游审批权限、开放中国公民在俄阿州境内72小时免签，增强黑河旅游吸引力。突出五大连池风景区国际矿泉水疗康养中心定位，谋划冰雪雨林、焦得布森林公园和火山主题乐园等旅游项目。丰富五大连池风景区夜文化，建设一批温泉、酒吧、咖啡厅等特色项目，利用现代技术策划神奇“水秀”，演绎景区神话传说，打造“北方小丽江”。发展冰雪经济，推进中俄冰雪主题乐园、卧牛湖冰雪旅游体验区、五大连池冰雪游乐区、逊克大平台雾凇冰雪摄影旅游区建设，逐步把黑河建成黑龙江特色冰雪旅游目的地。促进试车经济与冬季旅游融合，加快黑河汽车寒区试验基地建设，策划雪地漂移等冬季旅游产品，打造寒地试车城。与国内知名旅游创意企业合作，谋划旅游精品线路，重点打造爱辉—五大连池国际度假黄金旅游带、爱辉—孙吴—逊克百里界江中俄边境旅游带、嫩江—五大连池—北安驿站古道及现代农业乡村旅游带。组织好旅游产品营销活动，加大旅游纪念品开发力度。培育文化休闲产业。挖掘特色文化资源，突出欧陆、民俗、知青等特色文化元素，打造地域文化精品。谋划建设瑷珲—腾冲人口地理公园，加强瑷珲古城保护开发，铭刻历史文化记忆。吸引社会资本参与中俄民族风情园建设，丰富体验式活动内容，打造市域文化休闲首选地。加强非物质文化遗产保护，开发少数民族节庆活动，打造具有浓郁地方特色的休闲文化活动品牌。加快中俄宝石交流中心建设，推进俄罗斯珠宝玉石落地加工，开发逊克玛瑙、孙吴木化石和五大连池草化玉石，打造中俄宝石城，做大宝石产业。发展特色旅居养老产业。推动养老与康疗养生融合发展，加快黑河天沐龙福康疗养生养老广场、五大连池国际矿泉水疗康养中心项目建设，利用火山矿泉、矿泥和特殊地磁环境，吸引更多国内外老年人康疗养生，打造中俄跨国旅居养老和五大连池国际矿泉康疗养生品牌。创新医养结合服务模式，与旅居养老、康疗养生有机结合，谋划建设国际健康医学示范园区和跨境医养结合产业园区，提供高品质养老服务。

(六)彰显城市独特魅力，精心打造美好家园。城市承载着人们对美好生活的向往，坚持人民城市为人民，构建城乡新体系。完善城市功能。发挥规划引领作用，突出城市定位和地域特色，开展城市风貌规划研究，展现欧陆风情独特韵味。高起点谋划多功能国际会展中心，满足冬季举办大型展会需求，培育会展经济。规划建设停车场，改造环城路、龙滨路、王肃街人行道，实施公共自行车第二轮扩容升级，新增自行车站点70处、自行车1500辆，科学疏导交通，让市民出行更加方便。推进“三供三治”项目建设，市城区改造供水管网5.2公里、供热管网13.3公里，加快热源设备升级改造，新增供热面积35万平方米。推进城市管道燃气工程，新建燃气管道5公里、庭院管网6公里，庭院管网覆盖面达到5000户。优化城市环境。建设中俄双子城公园，推进黑龙江沿江公园改造，启动大黑河岛中心广场建设，为市民和游客打造亲水空间。实施大黑河岛植被还绿工程、电业街延伸路段绿化带建设工程，增加街头绿化、美化景观，提升城市品质。迎接好国家卫生城市复审工作。加强城市管理。建设智慧城市，完善数字化城管设施，推进信息资源整合共享。实行环卫、市政、园林市场化养护运行模式，确保市容市貌干净整洁。加强对市城区新建小区供水、供热、供电设施的监管，制定出台物业、房地产开发等城市管理条例。推进城管执法体制机制改革，理顺城市管理职能。协调推进城镇体系和美丽宜居乡村建设。强化市区、北安、嫩江3个区域中心城市功能，做大五大连池、孙吴、逊克3个秀美县城，做强“北五经济圈”，推进嫩江撤县建市，实现区域一体化发展。启动北安、逊克国家新型城镇化试点建设。打造瑷珲镇、通北镇、孙吴镇等特色小镇。合理规划乡村布局，开展环境卫生整治，重点提升建设41个村，打造特色鲜明的美丽宜居乡村示范带。

(七)加强生态建设，实现永续发展。用最严格的制度、最严厉的监管、最严肃的问责，保护好生态环境，构建生态新体系，造福子孙后代。保护好森林资源。完成人工造林1.4万亩、封山育林7.5万亩。做好中幼林抚育和林冠下补植，完成森林抚育任务37.2万亩。加快湿地公园建设，申报坤河国家级湿地公园。严厉打击“三毁”违法行为，严守森林、湿地资源红线。扩大嫩江西伯利亚杏生态经济试验林规模，促进沙化林地植被恢复。推进嫩江航站三期改扩建工程，争取森林火灾高危区综合治理项目立项，重启中俄森林防火联络站，严防重特大森林火灾。抓好国有林场改革，增强林业发展活力。强化环境治理。保护好干净的水，全面启动“河长制”河湖管理模式，加强水资源保护、水域岸线管理、水污染防治、水环境治理、水生态修复和涉河湖执法监管，形成人与自然和谐发展的河湖生态新格局。落实最严格水资源管理制度，确保考核位居全省前列。保护好优质的土，实施黑土地保护工程，大力推广农业“三减”技术，“三减”面积达到200万亩，推广绿色标准化栽培技术，面积达到800万亩。开展秸秆综合利用和禁烧工作，秸秆还田面积900万亩。保护好清新的空气，全部淘汰城市建成区内每小时10蒸吨以下燃煤锅炉，禁止新建每小时20蒸吨以下燃煤锅炉，提高空气质量监测水平，让“黑河蓝”成为引以自豪的常态。推进节能减排。积极应对资源稀缺、环境承载能力有限的现实挑战，走节约循环可持续发展之路。加快爱辉区国家级生态文明建设示范区创建步伐，力争省级生态市创建通过验收。加快循环经济发展，实施一批循环经济示范项目，支持有条件的县(市、区)、产业园区和企业开展循环经济试点建设。加强重点企业污染防治和已建成减排设施监管，建立环境监管数据信息库。严格节能目标考核，确保万元地区生产总值综合能耗按计划降低。

(八)坚持以人民为中心，增进群众福祉。牢固树立人民立场，把以人民为中心的发展思想体现在经济社会发展各个环节，构建社会治理新体系。坚决打赢脱贫攻坚战。把脱贫攻坚作为头等大事和第一民生工程，围绕“两不愁三保障”等刚性指标，多措并举，精准施策，做到真扶贫、真脱贫，全市省级贫困县(孙吴)摘帽，贫困村、贫困人口全部脱贫。实施基础建设扶贫，推进56个村整村扶贫攻坚工程。实施产业扶贫，加快贫困地区农民脱贫致富步伐。实施异地搬迁扶贫，推进贫困村屯人口向中心村集中。实施教育扶贫，让贫困地区的孩子接受良好教育，阻断贫困代际传递途径。实施健康扶贫，提高医药费报销比例，将因病致贫、返贫的贫困人口纳入农村低保、医疗救助范围。激发创新创业活力。坚持就业优先战略，以创新引领创业，以创业带动就业，实现零就业家庭动态清零。全市城镇新增就业1.4万人，城镇登记失业率控制在4.5%以内。加快孵化器、众创空间等创新创业服务平台建设，打造“创业苗圃—孵化器—加速器—产业园”创业孵化链条。把人才作为创新发展的第一要素，落实激励人才创新创业实施办法，推进人才管理体制改革，健全技能人才培养、评价和激励机制。完善产学研用合作培养人才模式，加快引进农业、林业、教育、科技、文化、卫生等领域急需紧缺人才，抓好省级领军人才梯队建设，促进人才智力项目对接。建立以需求为主导的对俄人才智力引进模式，重点引进急需的俄罗斯优秀人才、团队及智力成果。支持黑河学院建立“俄罗斯远东智库”。推动社会事业发展。实施“全面改薄”工程，改善贫困地区义务教育薄弱学校办学条件，完成62所学校新建、改扩建任务。提高义务教育均衡发展水平，创建标准化学校6所，市直和爱辉区完成国家义务教育发展基本均衡县建设任务，提升高中阶段教育质量。积极推动市委党校异地重建工作。办好老年大学。加大投入力度，构建现代公共文化服务体系。加快“健康黑河”建设，深化医药卫生体制改革，完善城乡医疗、公共卫生服务体系，提升群众健康指数。抓好复旦大学附属中山医院对市第一人民医院帮扶协作的技术落地，借助哈尔滨医科大学技术优势，搭建国际健康医学交流平台。市第一人民医院改扩建工程和儿科楼投入使用。开展丰富多彩的全民健身活动和竞技体育赛事，市全民健身中心体育馆投入使用。谋划建设群众冬季活动场所。完善社会保障体系。建立统一的城乡居民基本医疗保险制度，继续推进城镇职工(居民)基本医疗保险市级统筹，实施城镇职工医疗保险市级调剂金制度。做好机关事业单位养老保险制度改革实施工作。市精神病人福利院整体完工。维护社会安全稳定。完善安全生产信息化建设，打造互联网应用型安全监管体系。开展食品安全和药品流通领域专项整治，保障食品药品安全。推动以阳光信访、责任信访、法治信访、服务信访为目标的信访制度改革和法治化建设，维护群众合法权益。完善社区矫正数字化管理系统，实现全覆盖。推进“平安黑河”建设，全面构建立体化治安防控网络。深入开展法治宣传教育，全面启动“七五”普法工作。推进军政军民融合发展，加强科技管边、信息控边、合力治边，抓好反恐专业机构和队伍建设，维护边境秩序稳定。同时，进一步做好老区、民族宗教、防震减灾、侨务、妇女儿童、人防、档案、地方志等工作。

四、全面加强政府自身建设

新一届政府肩负着全面建成小康社会的神圣使命，承担着推进全面振兴发展的重要职责，必须倍加珍惜时代赋予的历史机遇，倍加珍惜全市人民的信任重托，用担当推动事业，用实绩赢得民心，努力建设人民满意政府。推进依法行政，打造法治型政府。把法治思维融入政府工作全过程，推进法治政府建设，纳入综合考核指标体系。坚决落实市委决策，自觉接受人大依法监督、政协民主监督，认真办理人大、政协建议和提案，虚心听取各民主党派、工商联、无党派人士和人民团体意见。普遍建立法律顾问制度，提高依法行政决策质量。委托第三方机构对重大行政决策开展评估论证，逐步实现专家信息和论证意见公开。健全重大行政决策风险评估机制，使评估结果成为决策的重要依据。加强能力建设，打造学习型政府。把强化学习作为政府工作硬任务，真正把读书学习当成一种生活态度、一种工作责任、一种精神追求，坚持政府班子常态化集中学习制度，深入开展政府系统专业知识专题学习活动，克服本领不足、本领恐慌、本领落后问题，在学习上下一番真功夫、实功夫、苦功夫，使专业素养和工作能力跟上时代节拍，成为做好工作的行家里手。注重理论联系实际，把理论知识、上级精神与黑河实际紧密结合，增强工作的科学性、预见性、主动性，用科学的方法解难题、谋突破。优化发展环境，打造效能型政府。把改革创新贯穿于政府工作各领域，加大“放管服”力度，深入推进行政审批、财税体制、商事制度、社会事业等重点领域改革。进一步减少审批事项，实现政府权责清单动态管理。理顺市直、爱辉区和边境经济合作区管理层级。做好事业单位分类改革工作，继续推进承担行政职能事业单位改革试点和生产经营类事业单位改革，清理规范市场中介机构。构建“亲”“清”新型政商关系，从严查处各类破坏经济发展环境行为。整合完善统一的公共资源交易监管平台。加快各县(市、区)行政权力流程梳理和网上审批平台建设。改进工作作风，打造责任型政府。把务实作风体现在政府工作各方面，各级政府班子成员带头深入基层，大兴调查研究之风，创新调研方式，畅通与人民群众密切联系渠道，准确把握问题的本质和规律，研究透彻解决问题的思路和对策。强化抓落实责任机制，对认定的事，全神贯注、紧盯不放，对定下的事，坚定不移、持之以恒，对已干的事，一抓到底、力求实效，努力锤炼干实事、能负责、敢作为、勇担当的优良作风。强化廉政勤政，打造廉洁型政府。把廉政勤政要求落实到政府工作各环节，市政府党组带头履行“一岗双责”，严格落实政府系统党风廉政建设主体责任。深入开展公共资源、政府采购、市政工程、国有资产等重点领域问题治理，坚决向懒政庸政不作为“亮剑”。跟踪督办重大决策执行情况，加强绩效管理、效能监察、审计监督和行政问责，确保重大决策部署贯彻落实，提高政府公信力和执行力，树立勤政为民、清正廉洁的政府形象。

各位代表，我们正处在全面建成小康社会、实现中华民族伟大复兴的关键时期，努力奋斗才能梦想成真。我们的信心来自于以习近平同志为核心的党中央的方向引领，来自于省委、省政府和市委的正确领导，来自于黑河改革开放探索实践和全市干部群众加快发展的强烈共识。在这个伟大的时代和宽广的舞台上，每个人都有足够的机会出彩。让我们在省委、省政府和市委的正确领导下，紧紧依靠全市各族人民，以只争朝夕的精神，撸起袖子加油干，提起创业精气神，为把黑河建设的更有活力、更有实力、更有魅力，开创更加幸福美好的新未来而努力奋斗！
